# Designing paper‐based records to improve the quality of nursing documentation in hospitals: A scoping review

**DOI:** 10.1111/jocn.15545

**Published:** 2020-11-07

**Authors:** Naomi Muinga, Ibukun‐Oluwa Omolade Abejirinde, Chris Paton, Mike English, Marjolein Zweekhorst

**Affiliations:** ^1^ Athena Institute VU University Amsterdam Amsterdam The Netherlands; ^2^ KEMRI/Wellcome Trust Research Programme Nairobi Kenya; ^3^ Department of Public Health Institute of Tropical Medicine Maternal and Reproductive Health Unit Antwerp Belgium; ^4^ International Program Evaluation Unit SickKids Centre for Global Child Health Toronto ON Canada; ^5^ Dalla Lana School of Public Health University of Toronto Toronto ON Canada; ^6^ Nuffield Department of Medicine Centre for Tropical Medicine and Global Health University of Oxford Oxford UK

**Keywords:** charting, documentation, inpatient, nursing records, observation charts, paper, review

## Abstract

**Background:**

Inpatient nursing documentation facilitates multi‐disciplinary team care and tracking of patient progress. In both high‐ and low‐ and middle‐income settings, it is largely paper‐based and may be used as a template for electronic medical records. However, there is limited evidence on how they have been developed.

**Objective:**

To synthesise evidence on how paper‐based nursing records have been developed and implemented in inpatient settings to support documentation of nursing care.

**Design:**

A scoping review guided by the Arksey and O'Malley framework and reported using PRISMA‐ScR guidelines.

**Eligibility criteria:**

We included studies that described the process of designing paper‐based inpatient records and excluded those focussing on electronic records. Included studies were published in English up to October 2019.

**Sources of evidence:**

PubMed, CINAHL, Web of Science and Cochrane supplemented by free‐text searches on Google Scholar and snowballing the reference sections of included papers.

**Results:**

12 studies met the eligibility criteria. We extracted data on study characteristics, the development process and outcomes related to documentation of inpatient care. Studies reviewed followed a process of problem identification, literature review, chart (re)design, piloting, implementation and evaluation but varied in their execution of each step. All studies except one reported a positive change in inpatient documentation or the adoption of charts amid various challenges.

**Conclusions:**

The approaches used seemed to work for each of the studies but could be strengthened by following a systematic process. Human‐centred Design provides a clear process that prioritises the healthcare professional's needs and their context to deliver a usable product. Problems with the chart could be addressed during the design phase rather than during implementation, thereby promoting chart ownership and uptake since users are involved throughout the design. This will translate to better documentation of inpatient care thus facilitating better patient tracking, improved team communication and better patient outcomes.

**Relevance to clinical practice:**

Paper‐based charts should be designed in a systematic and clear process that considers patient's and healthcare professional's needs contributing to improved uptake of charts and therefore better documentation.

**What does this paper contribute to the wider global clinical community?**

- Paper‐based charts for inpatient care have been developed following a general non‐systematic process of problem identification, literature review, chart (re)design, piloting, implementation and evaluation.
- The studies however executed each step differently leading to various documentation outcomes.
- This review proposes that a systematic process to chart design such as the human‐centred design approach might yield optimally designed charts that meet users' needs and lead to better documentation outcomes.

## 1 INTRODUCTION

Documentation of clinical care facilitates information flow between interdisciplinary healthcare providers, supports continuity of care for patients (Keenan et al., 2008) and supports the clinician's memory of care provided (Dalianis, 2018). Further, nursing care documentation serves objectives such as facilitating administrative processes that nurses perform, providing a formal legal document of nursing care provided, creating a record of care that can be used for education and providing data for quality improvement and research (Dalianis, 2018; Ioanna et al., 2007; Mann & Williams, 2003). Therefore, it is important to study the existing nursing documentation charts as their correct design and use could have significant implications for overall clinical management of patients (Urquhart et al., 2009).

While adoption of electronic patient records is progressing, paper continues to be an important medium for recording inpatient care in many settings and particularly in low‐ and middle‐income countries (LMIC). Even in high‐income settings such as the US, Australia and Europe, observation charts used to record daily patient physiological data such as vital signs are largely paper‐based (Cornish et al., 2019; Odell et al., 2009; Preece et al., 2012). Despite the dominance of paper as a medium for nursing records, research on their design is only beginning to emerge in high‐income settings (Christofidis et al., 2013; Isaacs et al., 2019; Preece et al., 2012). Structured and well‐designed paper records facilitate efficient data collection for quality monitoring purposes (Mwakyusa et al., 2006) and prepare the ground for future electronic medical records. Therefore, without careful design and implementation of paper‐based records in the first instance, the full benefits of computerisation are unlikely to be realised (Mann & Williams, 2003; Miller et al., 2010).

An evidence synthesis focussing on documenting nursing care found that aspects such as time spent documenting, documentation errors, legal accountability and interdisciplinary communication have been studied (Keenan et al., 2008). Cowden and Johnson (2004) found that many nursing admission forms in use were contributing to data duplication potentially hindering efforts for future computerisation. When data are collected multiple times, its integrity is compromised, contributing to inefficient use of limited resources. However, there is a paucity of literature on how paper‐based nursing records have been developed as part of efforts to improve the quality of documentation of inpatient care.

To fill this gap, this study aimed to synthesise evidence on how paper‐based nursing records have been developed within inpatient settings to support documentation of nursing care. Building an understanding of how these paper‐based records have been developed is important as it allows us to learn, compare and adopt methods that have been shown to work within our project in Kenya where documentation of inpatient paediatric care was found to be inadequate (Ogero et al., 2018) indicating the need for better charts. A scoping review was considered appropriate as we anticipated limited or poorly developed literature on the process of developing charts. We wanted to synthesise evidence from previous studies on the topic of interest, and we did not intend to do a meta‐analysis.

## 2 METHODS

The Arksey and O'Malley Framework (Arksey & O'Malley, 2005) for scoping reviews updated by Levac (Levac et al., 2010) was used to guide the review process. The PRISMA Extension for Scoping Reviews (PRISMA‐ScR) guidelines for reporting scoping reviews (Tricco et al., 2018) was adopted for reporting our results (Appendix S1). The protocol for this study was not registered in advance.

### 2.1 Conceptualisation of key terms

Four key terms, related synonyms and combinations were applied in developing the search syntax: ‘nursing care’, ‘documentation’, ‘inpatient’, and ‘quality improvement’. We focussed on care provided by nurses during the inpatient stay and the paper‐based nursing records used to document this care. We expected that relevant articles would be published as a quality improvement project and therefore included this term. A detailed description of the terms is provided in Table 1.

**Table 1:** Description of search terms and synonyms

Key term	Description	Search terms and synonyms
Documentation #1	Process of recording the details of patient care using either computerised information systems or paper‐based charts. Focus is on paper‐based charts only	The following words were used with the Boolean operator OR: checklists, charts, flow charts, job aids, decision aids, decision support tools, tools, instruments, protocol, guideline
Nursing care #2	The nursing process has been described as a 5‐sequential step process that guides nursing care. It involves assessment, diagnosis, planning, implementation, and evaluation (Toney‐Butler & Thayer, 2019). In the first step, subjective or objective data are required. Subjective data are verbal statements from patients or caregivers while objective data are measurable; data such as vital signs, intake and output, and height and weight (Toney‐Butler & Thayer, 2019). These objective measures are often repeated (depending on the severity of illness) at regular intervals throughout the admission until the patient is discharged. The objective assessment measures are systematically recorded to facilitate interprofessional team care between nursing staff and other cadres in the ward. For this scoping review, the focus is on studies that document objective measures of assessment	Combinations of the following words were used with the Boolean operator OR: Monitoring, assessment, numerical data, vital signs, input, output
Inpatient #3	A category of patients who are under observation in a hospital ward. These patients need repeated objective observations to be recorded for the duration of their stay	Combinations of the following words were used with the Boolean operator OR: Inpatient, hospitalisation, admitted, admission
Quality improvement #4	The process of improving paper‐based charts will likely be published as part of a quality improvement project which may be described in a variety of ways. These studies might refer to the process of developing a chart as well as the outcomes	Combinations of the following words were used with the Boolean operator OR: Quality improvement, practice improvement, before and after, develop, standardise
Filters		English and humans only

### 2.2 Search strategy

The terms used to build the search syntax were based on the following: Documentation AND Nursing care AND Inpatient AND Quality improvement. Related terms are presented in Table 1 and the detailed search strategy is provided in Appendix A.

### 2.3 Running the search

Between August and October 2019, and with the help of an information specialist, the search was constructed in PubMed and adapted to other databases including CINAHL, Web of Science and Cochrane. We included publications up to October 2019. Free‐text searches on Google Scholar and snowballing from the reference sections of included papers were conducted to supplement the search.

### 2.4 Study selection

**FIGURE 1:**
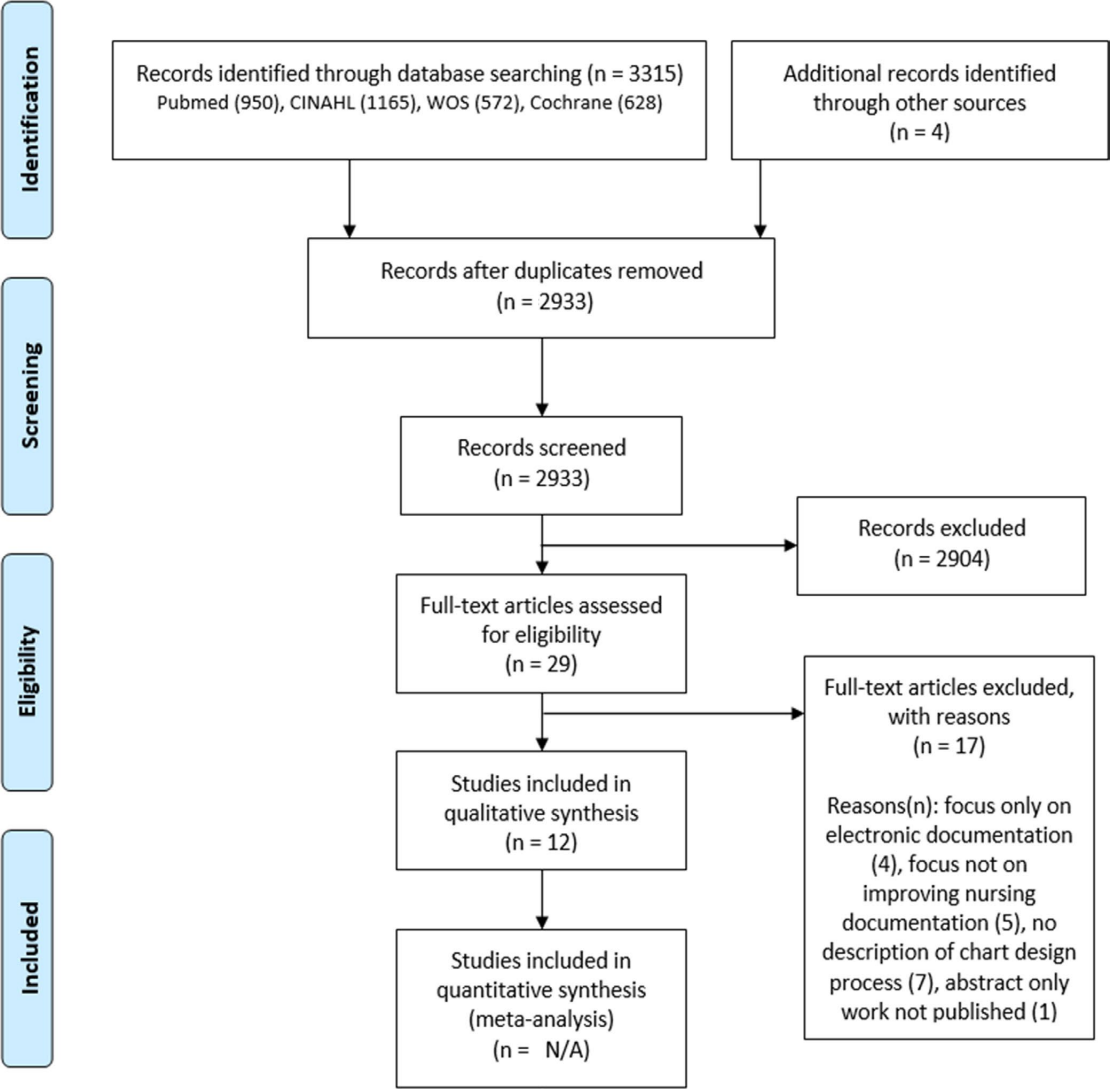
Flow of study selection [Colour figure can be viewed at wileyonlinelibrary.com]

All titles retrieved from the search were managed using Endnote X7.8 (Clarivate Analytics). Duplicate records were removed using Endnote duplicate function and by manual de‐duplication using Microsoft Excel. We adopted a two‐stage process for study selection. In the first stage, two researchers independently screened the titles and abstracts to identify studies for inclusion guided by two criteria: (1) Was the study about improving the quality of documentation? and (2) Was the study about nursing documentation for inpatient care (including emergency departments). We included emergency departments are these are likely to use similar observation charts to inpatient wards. We excluded studies that focused only on electronic documentation, those that designed charts specifically for nursing handover, and those whose purpose was to improve communication between staff rather than improve nursing documentation. In the second stage and before complete extraction commenced, one researcher scanned through the shortlisted articles to verify that they were appropriate for full data extraction. At this second stage, some papers were excluded for full data extraction for similar reasons as in stage one through consensus with two researchers as their suitability could not be established in the title and abstract screening phase. Figure 1 shows the flow of study selection.

### 2.5 Data extraction and analysis

A data extraction tool was developed by adapting and revising the Joana Briggs data extraction tool for scoping reviews (Joanna Briggs Institute, 2019) in discussion with the reviewers. Information extracted included general study information such as authors, year of study, country where the study was performed, hospital department and name of the chart. Other data extracted are described in Box 1. For quality assurance, full data extraction was performed independently by one researcher (all papers) and two researchers (6 papers each), ensuring that each paper was read and extracted by at least two authors.

**Guiding questions for data extraction**

1. Nature of the problem i.e. what led to the improvement or development of documentation charts?
2. The chart and its various components. E.g. were there other strategies that were part of the implementation apart from the new or improved or charts?
3. How were the paper‐based records developed or implemented? Did authors describe the process? To what extent was a co‐design or collaborative approach applied?
4. If available, what are the outcomes of the implementation? Description of what happened during or after implementation of charts.

Outcome of interest for this review were those directly related to documentation and not clinical outcomes. For example, number of times the new chart is used, or number of times items are documented on the chart.

1. What were the barriers and facilitators to implementing paper‐based records? What lessons were reported from the projects?
2. What recommendations do authors suggest when developing or improving paper charts?

## 3 RESULTS

12 studies were included in the analysis with all, except one conducted in high‐income countries. Majority of the studies were conducted in the USA (*n* = 5) followed by Australia (*n* = 3) and the United Kingdom (*n* = 2). New Zealand and Uganda had one study each. All studies were published between the year 1992 and 2017. The charts identified were either admission and/or discharge charts (Hill et al., 2014; North & Serkes, 1996; Okaisu et al., 2014; Street et al., 2017; Torakis & Smigielski, 2000; Vander Meer & Gabert, 1993) that capture one‐time events in the inpatient period, or were observation charts (also called flowsheets) (Cahill et al., 2011; Chatterjee et al., 2005; Gordon et al., 2008; Kuc, 2009; Robb & Seddon, 2010) that are used multiple times during the inpatient stay. The studies were descriptive case studies that employed a before and after study design. Where explicitly mentioned, the studies reported a non‐randomised prospective before and after intervention design (Cahill et al., 2011; Street et al., 2017) and an action research or cyclic methodology to design (Gordon et al., 2008; Hill et al., 2014; Okaisu et al., 2014).

The charts covered a range of clinical areas: adult surgical/medical or emergency care (Cahill et al., 2011; Gordon et al., 2008; Hill et al., 2014; Street et al., 2017), paediatric care (Okaisu et al., 2014; Torakis & Smigielski, 2000; Vander Meer & Gabert, 1993), and specialised seizure care (Kuc, 2009). In three studies, the charts covered all nursing units in the hospital (Chatterjee et al., 2005; North & Serkes, 1996; Robb & Seddon, 2010). We inferred the population to be adult, based on the cut‐off values of vital signs on the observation charts in two studies (Chatterjee et al., 2005; Robb & Seddon, 2010) and we found no nursing observation charts for newborn inpatient care. Uniquely, DiBlasi and Savage (1992) developed a complete documentation system comprising of: nursing admission assessment, a nursing care flowsheet and a re‐organised nursing care plan. An overview of the studies is provided in Table 2. The findings are presented in a narrative form as per the review questions (Box 1) in the next section.

**Table 2:** Overview of studies included and chart features

Author/year	Country	Setting	Target population or ward e.g. adult patients, newborns, etc.	Chart name and features	Funding
Street et al. (2017)	Australia	Three hospitals within one Australian metropolitan healthcare organisation.	Surgical patients admitted to the Post Anaesthesia Care units (PACU).	*Chart name*: Post‐Anaesthetic Care Tool (PACT) *General features (sample provided)* Number of pages: 3 (printed as a booklet) Orientation: Portrait Colour‐coded physiological signs with track and trigger system printed over 2 pages Abbreviations Tick boxes Plotting physiological values A legend on page 2 *Additional features/notes:* Clinical handover using ISOBAR (Introduction, Situation, Observation, Background, Assessment, Request) Discharge criteria Nursing notes section	Funded by the Health Contributions Fund Research Foundation
Hill et al. (2014)	Australia	A major metropolitan public teaching hospital.	Patients in the Oncology unit.	*Chart name*: Nursing oral and nutritional assessment tool *General features (sample provided)* Number of pages: 2 Orientation: Portrait Abbreviations Tick boxes A single A4 page with the oral assessment on the front and nutritional assessment on the rear Use of scores (0, 1, 2) for items plus total score for oral assessment Coding used for dietary requirements Weekly weights *Additional features/notes:* Used together with Malnutrition Universal Screening Tool (MUST) Refers to an oral hygiene policy when documenting	No statement made on funding.
Okaisu et al. (2014)	Uganda	A specialist paediatric neurosurgical teaching hospital.	Paediatric patients.	*Chart name*: Nursing admission and discharge record *General features (sample provided)* Structured Number of pages: 3 Orientation: Landscape Abbreviations Tick boxes *Additional features/notes* Items clustered according to sections: biodata, birth history, nutrition, home and community environment, family support, physical assessment and discharge information	No statement made on funding.
Cahill et al. (2011)	Australia	A university‐affiliated teaching hospital.	Surgical and medical ward patients.	*Chart name*: General observation chart *General features (sample provided)* Number of pages: 1 Orientation: Portrait Abbreviations Plotting physiological values (Blood Pressure, Pulse, Temperature) Neurological monitoring using AVPU scale Integration of the physiological triggers for the escalation plan Use of colour and sample provided in colour *Additional features/notes:* Blood pressure, pulse and temperature overlaid on one plotting area and differentiated using colour Respiratory rate elevated to the top of the chart	No statement made on funding.
Kuc (2009)	United Kingdom	A district general hospital.	All patients with seizure in the medical wards in the hospital.	*Chart name*: Fit Chart *General features (sample of page 1 only)* Number of pages: 2 Orientation: landscape Headings at the top limited to date, time, description and signature and a large space for repeated recordings of fit episodes Prompts consisting of a list of questions on the reverse side of the chart *Additional features/notes:* The absence of restrictive boxes suggested to the observer that he/she could record freely what had been seen	No statement made on funding.
Gordon et al. (2008)	USA	A tertiary care medical centre, a Level 1 trauma centre, and a National Cancer Institute.	Patients in 23 inpatient units and the emergency department.	*Chart name*: Daily nursing flowsheet *General features (sample of pain assessment table)* Instruction on how to fill the table Captures pain intervention and patient satisfaction Use of codes for pain reassessment with a legend provided at the bottom *Additional features/notes* The nursing flowsheet was modified to include pain assessment, but the rest of the flowsheet is not provided in the sample	No statement made on funding.
Robb and Seddon (2010)	New Zealand	A large metropolitan hospital.	Patients in the general medical and surgical wards, and specialist care units.	*Chart name*: Vital sign observation chart *General features (sample provided)* Number of pages: 1 Orientation: Portrait Abbreviations Vital signs: temperature, blood pressure, heart rate, and respiratory rate, each plotted on their scale Oxygen saturation, oxygen, glucose and pain score written as numbers Physically Unstable Patient (PUP) score section at the bottom Reported use of colour but sample provided in black and white *Additional features/notes:* Patient identification information affixed using a label	Counties Manukau District Health Board.
Chatterjee et al. (2005)	United Kingdom	A district general hospital.	Patients in all medical wards.	*Chart name*: Bedside observation chart *General features (sample provided)* Number of pages: 2 (printed on both sides) Orientation: portrait Temperature plotted on a large scale Blood pressure and heart rate overlaid on the same axis Pulse recorded as a number Respiratory rate plotted and written as a number Oxygen plotted on its scale A standard for detecting physiological decline—early warning score (EWS) at the back of the chart Warning lines corresponding to EWS score of 1 or 2 incorporated on plotted sections on page 1 *Additional features/notes* instruction on how to score and what action to take (e.g. inform nurse in charge) included at the back of the chart	None.
Torakis and Smigielski (2000)	USA	Children's Hospital of Michigan.	Paediatric patients admitted from the time of implementation.	*Chart name*: Paediatric admission database *General features (no sample provided)* 5 variables of the Neuman nursing process used as categories for assessment questions *Additional features/notes* A Neuman process summary document was developed to help incorporate the Neuman nursing process into nursing documentation. Sections include physiological, sociocultural, psychological, developmental and spiritual; all filled in free text.	No statement made on funding.
North and Serkes (1996)	USA	A regional medical centre.	Hospital‐wide (i.e. all patient groups).	*Chart name*: Hospital‐wide admission assessment form *General features (no sample provided)* Yes/No questions laid out in two columns Sections: Section 1: demographics and health history Section 2: clinical data Section 3: assessment of findings outside normal limits and formulation of a prioritised problem list *Additional features/notes* Additional assessments are documented on the 24 h flow sheet	No statement made on funding.
Vander Meer and Gabert (1993)	USA	15‐bed latency‐aged and 18‐bed adolescent units.	Children and adolescents.	*Chart name*: Nursing admission assessment tool *General features (partial sample provided)* Tick boxes Structured chart Space to provide a free‐text description Section 1: general admission information Section 2: organised under Gordon's 11 functional health patterns. Nursing diagnoses are listed under each pattern with space to add additional nursing diagnoses Problems requiring referral or consultations are listed under each pattern. *Additional features/notes* Space for prioritising nursing problems that need intervention within the next 24 h after review	No statement made on funding.
DiBlasi and Savage (1992)	USA	A free‐standing rehabilitation facility.	All patients admitted to the facility.	*Chart name*: A nursing admission assessment, A nursing care flowsheet, A nursing care plan (re‐organisation) *General features (no sample provided)* Gordon's Functional Health Patterns used as a framework for the admission assessment form Nursing care flowsheet: 7 of Gordon's Functional Health Patterns selected to organise information on the nursing care flowsheet The Uniform Data System (UDS) Functional Independence Measure (FIM) scoring system was used in selected functional areas to reflect the client's status *Additional features/notes* Sections to document client or family teaching included in the nursing care flow sheet	No statement made on funding.

### 3.1 Nature of problems leading to (re‐)design of charts

Where mentioned, the decision to improve paper charts in the inpatient setting originated from within the hospital or an external organisation. Within the hospitals, nursing departments or doctors identified challenges that needed to be addressed as (a) charts or documentation systems that were inadequate or that there was poor documentation of the care (DiBlasi & Savage, 1992; Hill et al., 2014; Okaisu et al., 2014; Vander Meer & Gabert, 1993); (b) a fragmented documentation system (DiBlasi & Savage, 1992); (c) incompatibility of existing documentation with new processes being introduced (Torakis & Smigielski, 2000); (d) poorly designed charts (Chatterjee et al., 2005; Kuc, 2009); (e) lack of specialised charts or multiple charts serving the same purpose (Kuc, 2009); e) outdated charts (Cahill et al., 2011); and (f) in response to multiple factors identified in the literature (such as failure to recognise clinical deterioration) (Robb & Seddon, 2010). In two studies, external organisations identified inefficiencies in the documentation that needed to be addressed to meet accreditation standards (Gordon et al., 2008; North & Serkes, 1996). The need for such standards was further supported by data gathered internally from the nursing departments.

### 3.2 Process of chart development

**FIGURE 2:**
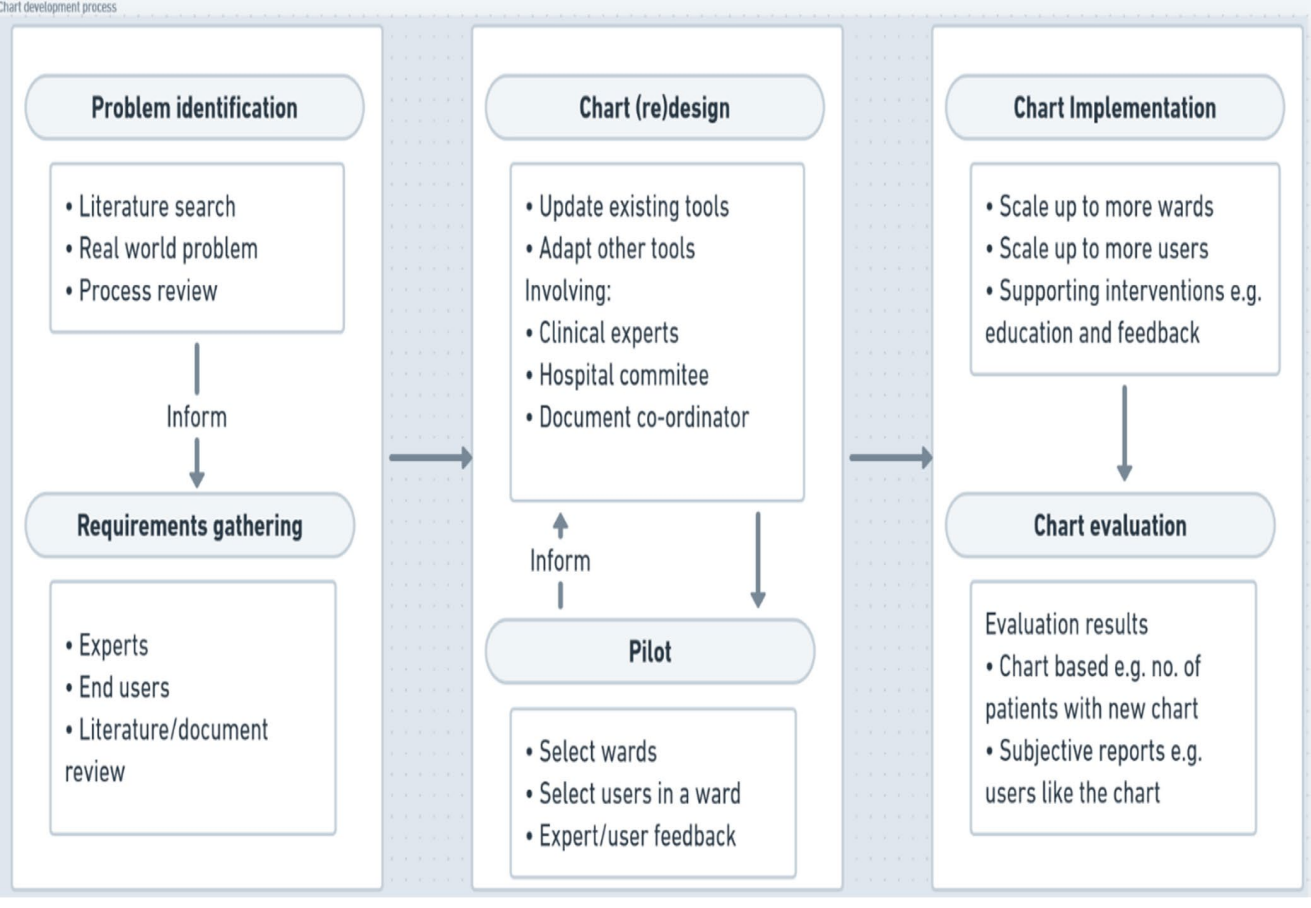
Chart development process [Colour figure can be viewed at wileyonlinelibrary.com]

Chart development followed a general process across the studies: problem identification and requirements gathering, chart (re)design and piloting, implementation and evaluation (Figure 2). Problem identification was done through a literature search as part of specific hospital research or quality improvement projects. For example, Street et al. (2017) conducted a detailed process review while DiBlasi and Savage (1992) examined the old system facilitated by a literature review. Next, the studies gathered requirements from experts and end‐users then developed a chart by re‐designing existing charts (Kuc, 2009; Torakis & Smigielski, 2000), adapting charts for the hospital context using findings from document and literature reviews (DiBlasi & Savage, 1992; Gordon et al., 2008; Hill et al., 2014; Street et al., 2017; Vander Meer & Gabert, 1993), and incorporating staff experience (Hill et al., 2014; Okaisu et al., 2014).

Various procedures were used to (re)design the charts. In two studies (Hill et al., 2014; Okaisu et al., 2014), researchers designed the chart with feedback from charge nurses, while in seven studies, a hospital committee or development group was constituted to design the chart in consultation with staff (Cahill et al., 2011; Gordon et al., 2008; North & Serkes, 1996; Robb & Seddon, 2010; Street et al., 2017; Torakis & Smigielski, 2000; Vander Meer & Gabert, 1993). In contrast, one study did not constitute a formal documentation committee but engaged groups of individuals at each phase of the design process (DiBlasi & Savage, 1992). In one study, a document coordinator was hired to ensure that the chart met accreditation standards (Vander Meer & Gabert, 1993). One study, that was addressing poor chart design, used findings from a simulation study combined with subjective chart preferences to design a new observation chart (Chatterjee et al., 2005). While studies used participatory approached to design, we found none that mention co‐design as an approach to designing the charts.

#### 3.2.1 Chart features

Six studies provided a full sample of the chart, three provided a partial chart while three only described the chart. Where full charts were available, the charts were commonly printed on both sides of A4 sheets in either portrait or landscape orientation. Two charts were three pages long; one printed as a booklet.

The observation charts plotted physiological data (temperature, heart rate, respiratory rate, blood pressure and oxygen saturation) and incorporated a colour‐coded early warning system overlaid on the chart (or provided on the reverse) to help in identification of out of range values (Cahill et al., 2011; Chatterjee et al., 2005; Robb & Seddon, 2010; Street et al., 2017). Chart plotting was implemented variably across the studies; for example, some used a combined scale for several vital signs while some used a separate scale for each vital sign. It is important to note however that most charts were downloaded in black and white and the colour‐coding was reported within the articles. The admission and discharge charts were highly standardised and therefore required minimal writing by using tick boxes and fixed options. In the two oldest studies, Gordon's Functional Health Patterns were incorporated into the nursing admission assessment chart (DiBlasi & Savage, 1992; Vander Meer & Gabert, 1993). Two charts by Kuc (2009) and Hill et al. (2014) were notably less structured than the observation charts; possibly to meet the need of detailed notes. A detailed description of chart features can be found in Table 2.

### 3.3 Chart piloting, re‐design and implementation

The next step in the design process involved piloting the charts in the clinical setting to obtain feedback from end‐users (Cahill et al., 2011; Kuc, 2009; Okaisu et al., 2014; Robb & Seddon, 2010; Torakis & Smigielski, 2000; Vander Meer & Gabert, 1993). These piloting and testing sessions revealed inadequacies around chart content and layout of the newly designed charts. For example, the first drafts of the charts by Okaisu et al. (2014) and Vander Meer and Gabert (1993) were found to be unnecessarily long, required large amounts of writing and the fold‐out format was not desirable. Following piloting, charts were re‐designed and implemented to additional wards or within the same ward on a larger scale. Lastly, an evaluation or chart audit was conducted.

Two studies, Vander Meer and Gabert (1993) and Hill et al. (2014), adopted a trainer of trainers (TOT) model to pilot and implement the chart while in the Cahill et al. (2011) study, a coordinator who was in contact with staff was identified. This review found that charts were often one part of a quality improvement project. Seven studies (Cahill et al., 2011; Gordon et al., 2008; Hill et al., 2014; Okaisu et al., 2014; Robb & Seddon, 2010; Torakis & Smigielski, 2000; Vander Meer & Gabert, 1993) developed and implemented the chart together with other strategies such as introducing a new assessment policy or a medical emergency response team while five (Chatterjee et al., 2005; DiBlasi & Savage, 1992; Kuc, 2009; North & Serkes, 1996; Street et al., 2017) focused on chart development and implementation.

To facilitate implementation, most studies trained staff on chart use (Cahill et al., 2011; Gordon et al., 2008; Hill et al., 2014; North & Serkes, 1996; Okaisu et al., 2014; Robb & Seddon, 2010; Torakis & Smigielski, 2000; Vander Meer & Gabert, 1993) while one study trained only ward sisters but no other staff (Kuc, 2009). Training programmes covered a range of issues including how to use the chart and education programmes specific to quality improvement projects. For example, where a process model was being introduced, staff also received training on the model (Torakis & Smigielski, 2000). Training was delivered via posters, presentations, meetings and written guidelines. To support implementation of charts, some studies instituted a policy or practise change (Gordon et al., 2008; Okaisu et al., 2014) while others improved how emergencies were identified (triggering mechanisms) by strengthening the emergency teams (Cahill et al., 2011; Robb & Seddon, 2010). Additional support during implementation was provided in some studies by conducting documentation compliance audits and giving feedback to nurses to stimulate documentation improvements (Gordon et al., 2008; Hill et al., 2014; Robb & Seddon, 2010).

### 3.4 Reported outcomes

#### 3.4.1 Documentation outcomes

For this review, we considered the primary outcomes as those related to documentation to allow for comparison. Documentation evaluation was carried out after 2–12 months of implementation with two studies repeating the evaluation; 5 months (Hill et al., 2014) and 3 years (North & Serkes, 1996). Seven studies reported better documentation measured by the number of new charts filled (Hill et al., 2014; North & Serkes, 1996), complete documentation of all vital signs (Cahill et al., 2011; Robb & Seddon, 2010), clinical assessment scores (Cahill et al., 2011; Robb & Seddon, 2010), pain management and adverse events (Gordon et al., 2008; Street et al., 2017). One study reported on improved accuracy of plotting vital signs (Chatterjee et al., 2005).

Of the remaining five studies, various measures of documentation outcomes were reported. One study reported decreased documentation time of more than 50% (DiBlasi & Savage, 1992), two reported that the charts enabled better tracking of patient progress (DiBlasi & Savage, 1992; Vander Meer & Gabert, 1993) while Okaisu et al. (2014) reported sustained improvement in the quality of nurses' assessment documentation. On the other hand, Torakis and Smigielski (2000) mentioned that staff experienced challenges filling the Neuman Process Summary form; this was a new process being introduced and therefore it might not have been fully understood. Lastly, Kuc (2009) reported poor adoption of seizure charts perhaps because only ward sisters were trained on how to use the chart, as opposed to all staff.

#### 3.4.2 Barriers and facilitators to implementation

The studies reported barriers and facilitators to chart development and implementation. Gordon et al. (2008) identified challenges related to process, people, policy and forms using an Ishikawa/fishbone diagram. The Fishbone diagram is a quality improvement tool for identifying problems and their causes (Ishikawa, 1976). Following this, they conducted an intensive 2‐week review which was not well received by the staff as it was perceived as being unnecessary. Knowledge deficit was a challenge when implementing the new programmes within which the new charts were being implemented (Robb & Seddon, 2010; Vander Meer & Gabert, 1993). Both studies conducted training and provided additional practise support to overcome the challenge. Hill et al. (2014) reported a lack of oral care equipment that hampered documentation; necessitating the commissioning of a mouthcare product trial.

Okaisu et al. (2014) attributed initial poor documentation to a cultural component of documentation practise that they corroborated by literature. They adopted a ‘system thinking’ approach to the improvement of nursing documentation which they believe contributed to achieving sustained improved documentation. Systems thinking is an approach to problem‐solving that considers relationships and interactions as part of elements that affect the problem (World Health Organization, 2009).The authors used this information to develop a new form, change hiring practises and improve the working environment.

Introducing a major design change to a section of the admission chart caused staff resistance during the initial implementation of admission chart reported by North and Serkes (1996). Nevertheless, following discussions, staff agreed to pilot the new form and found it easier to use it. Lastly, two studies suggested that successful implementation of charts requires staff involvement at all levels including the top level (DiBlasi & Savage, 1992; Vander Meer & Gabert, 1993).

## 4 DISCUSSION

### 4.1 Principle findings

This review aimed to synthesise evidence on how paper‐based nursing records have been developed and implemented in inpatient settings to support documentation of nursing care. From the evidence, studies reported developing paper‐based nursing records that were used once during the admission episode (admission and/or discharge charts) and those that were used multiple times to record patient progress (flowsheets or observation charts).

The studies reported varied methodologies in developing the charts beginning with problem identification and specification of the solution, followed by (re)design and piloting of the chart and finally implementation and evaluation of the charts. The drive to develop new charts came from within the hospitals or external accreditation organisations. In seven studies, the charts were developed and implemented together with other initiatives while five studies focused on chart development and implementation. All studies except one reported improved documentation outcomes: more new charts filled more of items filled in the chart, reduced documenting time, better plotting of physiological values and ability to get a better view of patient care or identify problems.

**FIGURE 3:**
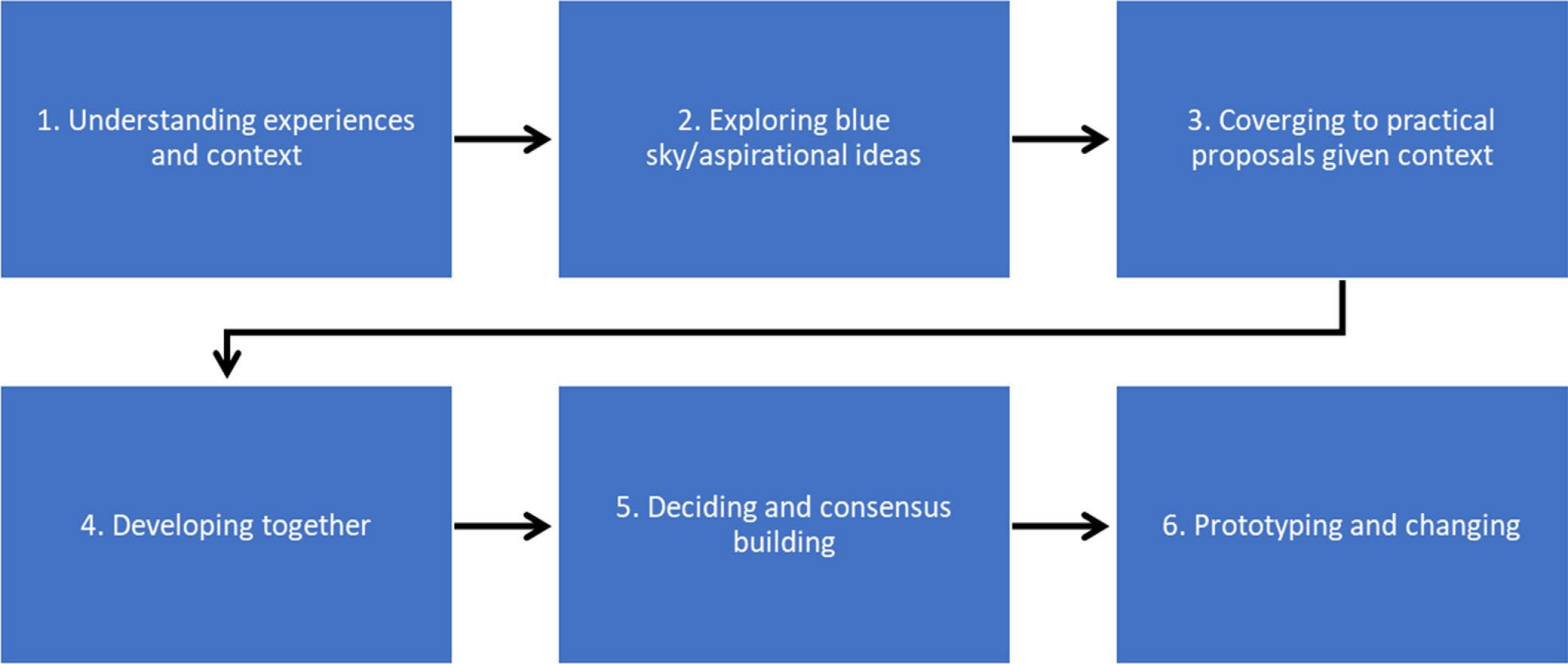
Adapted human‐centred design process [Colour figure can be viewed at wileyonlinelibrary.com]

Design problems identified during the piloting phase could have been averted or minimised by applying a systematic approach to chart design that considers the user's need and context. An example is the Human‐centred Design approach. This is an approach to developing interactive systems that focuses on the user, their needs and requirements by applying human factors/ergonomics techniques to improve user satisfaction, usability and sustainability of a product (International Organization for Standardization, 2019). The process has four major activities that occur iteratively: observation, idea generation, prototyping and testing (Norman, 2013). It follows a diamond model where designers go back and forth between generating diverse ideas and converging to workable solutions throughout the design process. To illustrate this, these four steps have been further expanded into 6 steps by various authors (Bowen et al., 2013; Boyd et al., 2012; Kim et al., 2019) beginning with understanding the experiences, exploring inspirational ideas, converging to practical proposals, developing together, consensus building and prototyping (Figure 3). The process is presented in a linear format, but one may go back and forth between stages as problems become clearer and new ideas emerge. A key emphasis of the Human‐centred Design approach is engagement with end‐users throughout the whole process.

In comparison, the studies in this review used literature reviews or an assessment of current practices to identify the problem and this would typically fall under step 1 of the Human‐centred Design process where one seeks to understand the user and the problem. However, the studies emphasised the problem rather than the user. More emphasis on the user would have enabled underlying issues such as knowledge deficit or team communication to be identified and addressed at design rather than implementation phase. This coupled with a systems approach to problem‐solving or other systematic quality improvement strategies such as the Fishbone approach provide opportunities to identify and solve problems. Steps 2–5 under the Human‐centred Design approach allow idea (including aspirational ideas), converging to practical proposals and developing together with users. The approach specifies a range of methods or tools that can be adopted to ensure that the users' needs are captured and addressed in the product design. In contrast, the studies reviewed tended towards receiving user feedback during the piloting and implementation phases. Finally, the Human‐centred Design approach includes a step to develop a prototype and change which can be compared to the piloting and re‐design phase reported by the studies.

The Human‐centred Design approach can be applied to both electronic and paper‐based products. As an illustration, Rogers et al. (2013) used a human factors approach applying the think‐aloud technique to evaluate system usability and identify barriers and facilitators to system use and inform re‐design opportunities for an electronic nursing information system. Likewise, the Australian Commission on Safety and Quality in Health Care (ACSQHC) is an example of an organisation that is making strides towards generating evidence on the design of observation and response charts. Work funded by the commission focussing on aspects such as recognising and responding to clinical deterioration (Horswill et al., 2010) aimed to develop an evidence‐based adult observation chart by evaluating current charts (Preece et al., 2009) and publishing a guide on how to design observation and response charts (Australian Commission on Safety & Quality in Health Care, 2019; Preece et al., 2010) that incorporates a human factors approach.

An important aspect of chart design is the content therein. Two nursing admission assessment charts in the review used Gordon's Functional Health Patterns to inform the content of the although the actual implementation differed. Similarly, for the nursing flowsheets, they were designed to monitor physiological signs but there was the varied implementation of early warning systems and numerical scales. This observed variability may be because there are no clear guidelines in the literature on how to design paper‐based medical records or how to report the design process. The Professional Record Standards Body in the UK develops and helps to implement standards for the structure and content of digital health and social care records ensuring a consistent and coherent approach to development and implementation of records that also facilitates information sharing (The PRSB, 2020). Developing a similar approach for paper‐based records, which may serve as templates for future electronic records, would ensure a consistent and comprehensive approach to documenting care where paper continues to dominate as a medium for recording care.

To evaluate the charts, the studies sought feedback from health professionals as well as assessed the chart use using various measures. However, there is an opportunity to conduct a systematic evaluation of charts using processes such as heuristics evaluation—a usability inspection method used in software development (Nielsen, 1994b). Heuristics evaluation is a method for identifying usability problems in an interface so that they can be addressed during the design period Nielsen (1994a). The method borrows from principles in human‐computer interaction and can be applied to any interface that requires human interaction including paper‐based charts. Preece et al. (2009) conducted a heuristic evaluation of 25 adult observation charts from Australia and New Zealand to improve management of deteriorating patients by improving the design of charts. Their evaluation identified 1189 usability problems to do with chart and content layout among other issues and these would inform usability principles related to paper‐based charts.

The limited body of work around the systematic, evidence‐based design of paper‐based charts has so far originated from high‐income countries but provides a starting point to developing charts. We suggest that more studies are required in low‐income settings so contextual differences can be identified and addressed. To contribute to this growing body of work, we are developing an inpatient newborn observation chart using the Human‐centred design approach to meet the need of better monitoring charts in LMIC. Additionally, we suggest that further work explores development of a systematic guide to designing and reporting on paper‐based charts be conducted.

### 4.2 Limitations

We found limited published literature that would allow us to answer our study question comprehensively. Majority of the studies excluded in the study selection phase were studies that focused on electronic documentation systems or that sought to evaluate already designed charts with no reference to the design process that we were interested in. As there are no published reporting guidelines on how to report these types of studies, the studies identified in this review were diverse in both the process of developing the chart as well as reporting. This made it difficult to compare the studies and draw general conclusions on what would be the best process to follow when designing nursing charts. However, this is not surprising as the literature in this area is only beginning to emerge. Despite these limitations, we believe that the evidence generated by this review contributes to the literature by highlighting current efforts to improve paper‐based nursing records as well as suggest opportunities for new studies.

## 5 CONCLUSIONS

This review presents evidence on how charts for documenting inpatient nursing care have been developed. The studies follow a general process of problem identification, literature review, chart (re)design, piloting, implementation and evaluation with varied execution of each step and a range of outcomes regarding improved documentation. The approaches used are like those outlined in human‐centred design: observation, idea generation, prototyping and testing. The Human‐centred Design approach puts emphasis on the user, their needs and experience to deliver usable products. While this approach is not the only method the authors could have used for their chart design, adherence to all the steps would have strengthened the design process and perhaps lead to better adoption of charts. Additionally, other issues such as lack of knowledge by health professionals and team dynamics as may have been identified early by adopting a systems thinking approach to chart development. We suggest that further work exploring the development of a systematic guide to developing and reporting on paper‐based charts be conducted.

## 6 RELEVANCE TO CLINICAL PRACTICE

Paper‐based charts should be designed in a systematic and clear process that considers patient's and healthcare professional's needs. This study has identified gaps in the process of designing observation charts for inpatient care and suggests the Human‐centred Design approach as a systematic process to design for better documentation outcomes. Using the Human‐centred Design approach provides an opportunity to address problems with the chart during the design phase as well as meeting the health professional's needs. This in turn promotes ownership and uptake because the users are involved at all stages of design. With improved uptake of charts, this will translate to better documentation of monitoring care thereby allowing health professionals to track patient progress, facilitate team communication, tailor care and achieve better patient outcomes.

## CONFLICT OF INTEREST

The authors declare that they have no conflict of interests.

## AUTHOR CONTRIBUTIONS

Study design: all authors; data collection and analysis: NM, IOA, CP; and manuscript preparation and revisions: NM, IOA, CP, ME, MZ. All authors approved the final version of the manuscript.

## ETHICAL APPROVAL

Ethical approval was not required for this paper.

## Supporting information

Appendix S1

## Appendix A

### Search strategy

Search run on PubMed on 28 08 19 and translated to other databases with the help of an information specialist.

**Table:**

Concept	Search
Documentation #1	TI ‘observation chart*’ OR AB ‘observation chart*’ OR TI worksheet* OR AB worksheet*) OR TI checklist* OR AB checklist* OR (MH ‘Checklists’) OR TI Kardex* OR AB Kardex* OR TI cardex* OR AB cardex* OR TI flowchart* OR AB flowchart* OR (MH ‘Documentation+’) OR TI (documentation OR documenting) OR AB (documentation OR documenting) OR TI records OR AB records OR TI protocol OR AB protocol OR (MH ‘Nursing Protocols+’) OR TI ‘job aid*’ OR AB ‘job aid*’ OR TI ‘decision aid*’ OR AB ‘decision aid*’ OR TI ‘decision support tool*’ OR AB ‘decision support tool*’ OR TI guideline* OR AB guideline* OR TI careplan* OR AB careplan* OR TI instrument OR AB instrument OR (MH "Nursing Records")
Nursing care #2	(MH ‘Monitoring, Physiologic+’) OR TI ‘nursing process*’ OR AB ‘nursing process*’ OR TI ‘nursing assessment*’ OR AB ‘nursing assessment*’ OR (MH ‘Nursing Assessment’) OR TI ‘vital signs’ OR AB ‘vital signs’ OR TI ‘monitoring output*’ OR AB ‘monitoring output*’ OR TI ‘monitoring input*’ OR AB ‘monitoring input*’ OR (MH ‘Nursing Process+’)
Inpatient #3	TI inpatient* OR AB inpatient* OR (MH ‘Inpatients’) OR TI hospitalisation OR AB hospitalisation OR TI hospitalisation OR AB hospitalisation OR TI admitted OR AB admitted OR TI admission* OR AB admission*
Quality improvement #4	(MH ‘Quality Improvement+’) OR TI ‘quality improvement*’ OR AB ‘quality improvement*’ OR TI ‘practice improvement*’ OR AB ‘practice improvement*’ OR TI (before AND after) OR AB (before AND after) OR TI (improve* OR develop* OR standard*) OR AB (improve* OR develop* OR standard*)
	#1 AND #2 AND #3 AND #4 AND ENGLISH AND HUMANS
